# Food Safety Awareness and Opinions in China: A Social Network Analysis Approach

**DOI:** 10.3390/foods11182909

**Published:** 2022-09-19

**Authors:** Lei Xia, Bo Chen, Kyle Hunt, Jun Zhuang, Cen Song

**Affiliations:** 1 State-Owned Assets Management Department, Nanjing Forestry University, Nanjing 210037, China; 2 School of Economics and Management, China University of Petroleum, Beijing 102200, China; 3 Department of Management Science and Systems, University at Buffalo, Buffalo, NY 14260, USA; 4 Department of Industrial and Systems Engineering, University at Buffalo, Buffalo, NY 14260, USA

**Keywords:** food safety, social network analysis, public opinion awareness

## Abstract

Over recent years, food safety has garnered widespread attention and concern from society. Concurrently, social media sites and online forums have become popular platforms to disseminate news, share opinions, and connect with one’s social network. In this research, we focus on the intersection of food safety and online social networking by utilizing natural language processing techniques and social network analysis to study public opinions related to food safety. Using real data collected from a popular Chinese question-and-answer platform, we first identify hot topics related to food safety, and then analyze the emotional state of users in each community (i.e., users communicating about the same topic) to understand the public’s sentiment related to different food safety topics. We proceed by forming semantic networks to analyze the characteristics of food safety opinion networks. Our results show that Internet users form modular communities, each with differences in topics of concern and emotional states of community users. Users focus on a wide range of topics, showing that overall, food safety awareness is increasing. This paper provides novel insights that can help interested stakeholders monitor the discussions and opinions related to food safety.

## 1. Introduction and Literature Review

According to the 49th Statistical Report on the Development Status of the Internet in China released by the China Internet Network Information Center, as of December 2021, there were 1.032 billion Internet users in China. With 42.96 million new Internet users compared to December 2020, the Internet penetration rate reached 73%, an increase of 2.6 percentage points compared to December 2020. The per capita weekly online time is 26.9 h for Chinese citizens [1], and similar trends in Internet usage can be found throughout the world. Along with the rise of Internet activity, social media has become an important medium for the general public to express their attitudes and views and has played an important role in the development and dissemination of public opinions. The connections formed by social media friends, followers, and comments trigger the formation of social networks. Opinion leaders in social networks have a heavy impact on the community, and their published contents are likely to influence the general users’ opinions to a certain extent and guide the direction of online discussion and public opinion.

Due to the many illnesses that can stem from unsanitary or contaminated foods, along with the global scale of food supply chains, food safety has become an important issue in today’s society. Given the rapid dissemination of information that the online environment allows, many people have turned to social media sites and discussion forums to discuss issues related to food safety. In view of this phenomenon, in this work, we adopt techniques from natural language processing and social network analysis to study the opinions surrounding food safety. Combining social network analysis with public opinion analysis, a social network opinion model is constructed, which is used for providing a comprehensive analysis of social network relationships, popular topics, and the emotional states of social network users who discuss issues related to food safety. Focusing on the opinion leaders in virtual communities and the differentiation between communities, we analyze public conversations related to food safety, which provides insights into community management and user relationship management on social media.

### 1.1. Social Network Analysis Research

Social network analysis (SNA) is a method that focuses on patterns of relationships among people, as well as among groups such as organizations and states. SNA techniques can deal with large amounts of relational data to describe overall network structures and features, such as the most important nodes (users) and the strongest connections (relationships) within a network [2]. SNA encompasses a range of techniques for mapping, measuring, and analyzing social networks by using both qualitative and quantitative data [3]. Some of the common metrics that are studied in the SNA literature include the degree of entry (i.e., the in-degree of a node), degree of exit (i.e., the out-degree of a node), centrality, and network density.

In the current environment of the rapid development of social media, existing studies focus on the formation of user communities and the spread of public opinion within social network platforms. Based on mathematical models and machine learning algorithms, the behavioral and emotional characteristics of community users can be analyzed to monitor the hot spots and directions of public opinion in online communities. Yao et al. (2021) applied social network analysis to investigate knowledge sharing on Twitter to find opinion leaders and carried out cluster analysis, sentiment analysis, and relevance analysis on the collected tweets [4]. Using social network data from Twitter and Colab (a Brazilian social networking site to connect citizens with the government), Benke et al. (2020) applied machine learning algorithms to automatically classify citizens’ messages according to different urban service dimensions [5].

Meanwhile, social network analysis has also been applied in the fields of public health, economic development, security science, and sustainable development. Poudyal et al. (2021) used social cognitive career theory and UCINET software V6.232(Harvard, Cambridge, MA, USA) to analyze whether the social network attributes affect the academic productivity of researchers [6]. Reyhani and Grundman (2021) applied participatory stakeholder analysis and social network analysis to identify stakeholders in the water governance system and analyzed their attributes and network characteristics [7]. Praet et al. (2021) analyzed the network of relationships among parliamentarians based on Twitter data [8]. Bertoni et al. (2022) obtained questionnaires and structured interview data from hospitals to identify key players contributing to resilient healthcare [9]. Gongora-Svartzman and Ramirez-Marquez (2021) combined textual and graphical network analysis to investigate how social cohesion can be quantified through social media channels during disasters [10].

### 1.2. Social Media-Based Public Opinion Analysis

The advent of social media has led to a plethora of social network data available for sentiment analysis and related research. For example, Melton et al. (2021) collected Reddit community text data for sentiment analysis to monitor changes in user sentiment and opinion topics [11]. Based on the VADER dictionary and LDA, Ridhwan and Hargreaves (2021) conducted an overall sentiment analysis and thematic analysis of tweets related to COVID-19 in Singapore [12].

A variety of text analysis methods are used in the field of public opinion analysis to better understand the topics and sentiment tendencies. Tu and Huang (2016) proposed a framework for mining and analyzing personal interests from microblogging texts, using a new algorithm that combines word frequency-inverse document frequency (TF-IDF) with TextRank to rank each keyword [13]. Hu et al. (2018) proposed an online Biterm Topic Model (BTM) based approach to refine short text stream classification [14]. He et al. (2020) used TextRank and LDA to study the topic distribution of technology supply and demand [15]. Feldmeyer and Johnson (2022) collected spontaneous consumer comments about turmeric on Twitter and used BTM topic modeling to analyze consumer opinions [16]. In Song et al. (2020), the authors used LDA and k-means to extract food safety topics from Sina Weibo discussions and then studied the sentiment of the users who posted about food safety [17].

There is also a stream of research that is focused on improving the accuracy of text analysis techniques by comparing and optimizing existing algorithms. Vargas-Calderón and Camargo (2019) proposed a method to extract communities on Twitter by deploying a clustering algorithm to mine potential topics, where Word2Vec models and natural language processing techniques were used to represent the complete set of tweets [18]. Alam et al. (2020) applied domain-specific distributed word representation (DS-DWR) and convolutional neural networks to conduct sentiment analysis [19]. By using four classifiers, including plain Bayesian, decision tree, random forest, and support vector machine, Neogi et al. (2021) used Bag of Words and TF-IDF to convert Twitter text messages into numerical weights in vector format for prediction [20]. After collecting random subsets of all tweets containing geographic information through Twitter’s application programming interface (API), Huerta et al. (2021) conducted emotional analysis and language analysis using the VADER model and language query and word count statistical tool and evaluated the effect of public health interventions by using intermittent time series (ITS) analysis [21].

### 1.3. Public Opinion Analysis on Food Safety

Based on the original food safety opinion models, new algorithms and metrics have improved the accuracy of food safety opinion monitoring. For example, Chen and Zhang (2022) proposed a transfer learning-based approach, the BERT-based Alternative Meat (BAM) model, to explore public attitudes toward alternative meats [22]. With the gradual increase in the size of Internet users, social media platforms such as Facebook, Twitter, and YouTube have become the best choice for collecting data, public opinions, discussions, and online questionnaires [23]. Wu (2015) used web surveys, multiple regression analysis, and Fuzzy set qualitative comparative analysis (FSQCA) to examine the risk communication strategy of Facebook in the context of food safety issues [24]. Singh et al. (2018) used Twitter data to identify supply chain management issues in the food industry using support vector machine (SVM) text analysis and hierarchical clustering with multiscale bootstrap resampling [25]. By collecting user posts on social networking sites such as Twitter, Facebook, and Instagram, Guzik et al. (2022) conducted sentiment analysis to discern consumer perceptions of microwavable foods [26].

As food safety is a key aspect of public health, there is a plethora of research focusing on analyzing public opinions related to food safety issues. However, this literature is methodologically limited to the natural language process and machine learning techniques that have been discussed thus far. To the best of our knowledge, there is a dearth of research that has applied social network analysis to study food safety. In order to fill the gaps in the literature, this paper combines social network analysis with public opinion analysis, where we study the role of opinion leaders and the evolution of public opinions in different online communities (where a community represents a set of users discussing a specific topic), and focuses on the differences among communities.

## 2. Model Design of Social Network Opinion Analysis Related to Food Safety

### 2.1. Data Source and Processing

The data in this study are collected from Zhihu, which is a popular Chinese question-and-answer (Q&A) platform that has millions of active monthly users. We collected Q&A data from 2020–2022 under the topic of “food safety”, where the user data included user IDs, locations, genders, occupations, and the text of the posts. When a user posts a question in Zhihu, “answer users” answer that question directly in the system. Based on the answer, “comment users” can comment on the answer, even with another question. According to the new question within the content of the answer, “reply users” would further answer that question. In general, the name of the question user posting the initial question is not displayed on the platform. Therefore, the data chain is as follows: “answer users-comment users-reply users”. 

Our initial data collection resulted in a total of 28,936 crawled data chains, and after removing duplicate values and irrelevant data, 15,070 valid data chains were obtained, consisting of 30,006 total posts.

In this research, the data are pre-processed for all text analysis tasks as follows: (1) word separation of text data by the Jieba word separation tool; (2) elimination of deactivated words in the documents after word separation according to the deactivation word list [27]; (3) filtering of special texts such as emoticons and web links.

### 2.2. Flowchart of Social Network Opinion Analysis

**Figure 1:**
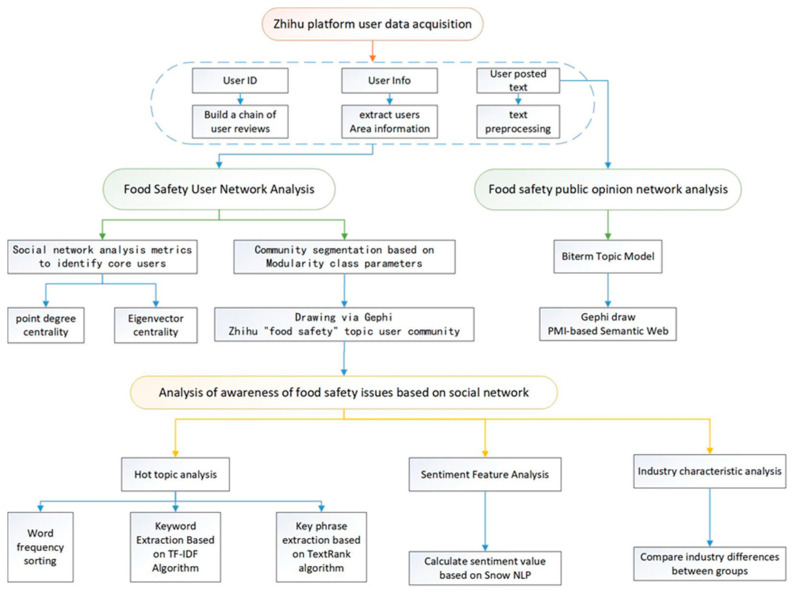
The methodological approach followed in this study.

The flowchart in Figure 1 shows the methodology that we followed in this research. We select hot subtopics under the main topic of “food safety” on the Zhihu platform and use web crawling technology to obtain user data and published content. Based on Wang et al. (2021) [28] and Xing and Li (2020) [29], the retweeting, commenting, and liking behaviors of social network users reflect the existence of relationship networks. Therefore, this study uses user IDs to establish comment relationship chains. Most of the existing studies use natural language processing to analyze the content of social platform posts, such as Featherstone et al. (2020) [30] using the semantic web and Jabalameli et al. (2022) [31] using topic modeling and sentiment analysis to process Twitter data. In all, the current study uses multiple methods to analyze public opinions using text-based data from online postings.

This research is generally divided into two parts, wherein in the first part, we create and analyze user networks using methods from the social network analysis domain, and in the second part, we develop and analyze public opinion networks. More specifically, in the first part of this research, we focus on the analysis of food safety user networks, where these networks are built according to the data chains that we collected from Zhihu. In analyzing these networks, degree centrality and eigenvector centrality are important metrics that can help identify key users in the group. Modularity is used to measure the strength of community divisions. A strong division is exhibited when communities that are formed have a high similarity between nodes (i.e., the nodes in a community share common features), while the similarity of nodes outside the respective communities is low. For this task, Gephi software is used to perform modularity calculations using the community discovery algorithm (i.e., the Louvain algorithm) to calculate the parameters of the modularity class. Users who are divided into the same community have the same parameters. Based on this division, the user network is partitioned into multiple communities, and the user community map can be drawn and analyzed.

Next, the public opinion analysis is performed on user texts in different communities, consisting of the following steps: (1) hot topic analysis, where we (i) sort each group of keywords by word frequency, (ii) extract keyword phrases based on the TF-IDF algorithm, and (iii) extract key phrases and abstracts based on the TextRank algorithm; (2) sentiment analysis, where we use the Python SnowNLP library to calculate values for the overall user sentiment, and each community’s sentiment; and (3) analysis of industry characteristics where the industry of platform users and the industry distribution in each community are summarized to analyze the overall distribution of platform users. 

The second part of this research focuses on the network analysis of food safety public opinions. In this study, the BTM is selected to identify the main topics of discussion related to food safety on the Zhihu platform. The point mutual information (PMI) index is often used to measure the correlation between two words in data mining or information retrieval and can therefore be used as the edge weight between two words (nodes) in a network. The inter-word edge weights are imported into Gephi software V0.9.2 (Gephi, London, UK) to draw the semantic network. After identifying the keywords through degree centrality, the topics are then identified through the community division algorithm.

## 3. User Network Analysis

### 3.1. Identification of Core Users

In this study, we develop networks where the nodes are represented by unique Zhihu users, and the edges are represented by comment relationships. More specifically, if user B comments or replies to user A, a directed edge from node A to node B is formed in the graph. The number of nodes in our graph is 2.349, the number of edges is 7.797, and the average user degree is 3.319, which indicates that the nodes (users) in the network are connected to 3.319 users on average. The network diameter is 3, indicating that the longest user chain is 3. 

In-degree in social networks measures the amount of information flowing from other nodes to the target node, and out-degree measures the amount of information flowing from the target node to other nodes. The degree of a node is the sum of its out-degree and in-degree (i.e., the degree of a node is the number of edges that are incident to that node) [32]. The number of other nodes that are directly connected to a target node can be used to identify core users in the community of users. In line with this concept, degree centrality is the most direct measure of node centrality in network analysis and is simply calculated as the degree of a node. The greater the degree of centrality of a node, the more important the node is in the network.

Table 1 shows the user relationship degree centrality statistics of Zhihu’s “food safety” topic. “Tahini,” “Qian Cheng,” and “milk” have the highest degree centralities, where their degrees are 1099, 462, and 404, respectively. It means that these users have a high degree of participation under the topic of “food safety,” where the users with a high degree of centrality represent the core users in the community.

Table 2 shows the statistics of edge weight in the user relationship graph. The node-weighted degree not only considers the weight of the edge but also considers the node degree. The weight of the user edge refers to the number of times two nodes are connected in the chain.

The eigenvector centrality measures the importance of the users connected to a specific user, and the value ranges from 0 to 1. As shown in Table 3, the top five eigenvector centralities in the user relationship graph are “rice grains,” “Suifeng,” “Nicole,” “Kratos,” and “Melon Eaters”.

The more important adjacent nodes of a node are, the eigenvector centrality of the node is higher. This metric shows that users with high eigenvector centrality have commented on users with high degree centrality on the platform, which indicates that users with high degree centrality are key users of the platform (i.e., they are influential users).

The degree centrality of user “rice grains” is 19, and the degree centrality of “Suifeng” and “Nicole” is 7. These three users commented on the important users such as “Tahini,” “zhihu user 0xyqjw,” “wang SH,” “zhihu user wp94Wr,” “Dongjun,” “Xiao Chubai,” and “Boy Sleep”. As an active user of the platform, “Boy Sleep” frequently comments on other important users, so its eigenvector centrality is also high. Although “Tahini” is a key user of the platform, it is connected and mostly commented on by ordinary users and does not comment on other core users; therefore, its eigenvector centrality is low. 

### 3.2. Community Division

**Figure 2:**
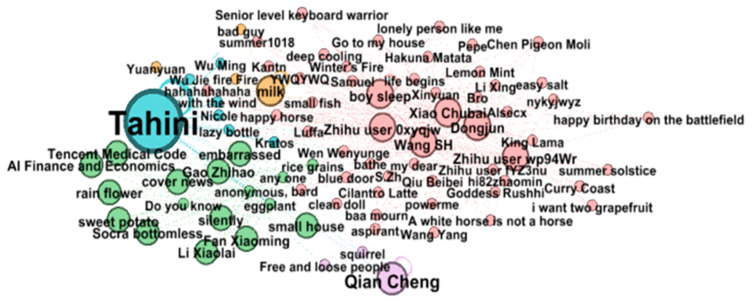
User community structure on food safety.

Community detection, or community discovery, reveals the aggregation behavior of social networks through network clustering. This paper applies the ForceAtlas2 layout and community detection algorithm to modularize the nodes into several communities. The modularity degree takes values between 0 and 1, and the larger the modularity index is, the more accurate the community structure of the network division is, and the modularity index generally appears between 0.3 and 0.7 in reality [33]. The modularity index is calculated as 0.662, which means it possesses small-worldness. After calculating the modularity class parameter, small groups (or small communities) can be segmented, and the community aggregation network will be formed after screening out some nodes (see Figure 2). The user community division is sorted by degree centrality. It can be seen that each group has core users in the community, and the core users are the center of the group, forming a virtual community.

### 3.3. Analysis of Food Safety Awareness Based on User Community Division

#### 3.3.1. Hot Topic Analysis

This section groups the data posted by users from different communities, which are divided in Section 3.2, and selects various methods to extract key information from the text to identify the hot topics of interest within each group.

(1) Hot topics based on word frequency

After text content sorting, the hot topics based on the word frequency discussed by each group of users are shown in Table 4.

The first group of terms with high frequency includes “pickle,” “company,” “problem,” and “food,” among others. Users in Group 1 are concerned about the production hygiene problem of “Laotan pickled cabbage,” which was exposed in the CCTV “3-15” evening party in 2022 (the 3-15 evening party is a public welfare event co-hosted and broadcasted live on the evening of 15 March every year by the China Central Radio and Television Station and national government departments to safeguard consumer rights and interests). The company mainly processes pickle packages for many famous brands. According to the Food Safety Law of the People’s Republic of China, food producers should check the supplier’s license and product certification; food ingredients that cannot provide certification should be tested in accordance with food safety standards. However, CHAQICAIYE company (Yueyang, China) said it would not test the health indicators of production raw materials of pothole pickled cabbage, and its production environment is not in line with food hygiene standards. 

Netizens, who are people actively involved in online communities, express their anger and panic over this shocking food safety incident, especially for the national brands Master Kong and Tongyi, which both have large sales volumes and wide distribution ranges. Under this topic, many other internet users talked about similar food safety problems with other brands, disappointed that national food safety is not yet guaranteed. As for “China” and “abroad” appearing in the top 10 terms for Group 1, this is due to the fact that CHAQICAIYE exports its high-quality products abroad while selling the harmful pot hole pickled cabbage to domestic food companies, which carries serious health concerns. This practice is due to the fact that the fines for domestic food safety problems are between CNY 1000 and 2000, while the fines are at least CNY 100,000 abroad. This kind of behavior causes strong dissatisfaction among internet users.

The second group of words that appear most frequently are largely related to fats and cooking oils, while high-frequency words include “peanut oil,” “olive oil,” and “blend oil”. This demonstrates that the community is concerned about whether the various types of oils and fats that are used in food preparation are healthy. At the same time, terms such as “smoke point,” “GMO,” and “oleic acid” appear many times in the discussion, indicating that the discussion of food safety topics tends to be more specialized in this group.

The third group of the network groups focuses on the topic of “Weidendorf milk exposed by CCTV as a fake foreign brand”. As early as 5 January 2016, CCTV exposed the fact that the brand of Weidendorf milk is registered in Germany and fully supported by Chinese companies, but it has never been sold in Germany. It is interesting that Weidendorf milk is still sold in large quantities on Jingdong, Tmall, and other e-commerce platforms. Most of the replies from users on the Zhihu platform prefer Weidendorf milk because its milk source and production are from Europe. They also comment that German milk not only solves the problem regarding people’s worry about the quality of domestic milk but also satisfies the patriotic feeling of people who want to support national products. “Mengniu”, “Yili”, “domestic”, and “delicious” show the comparison between Weidendorf milk and domestic milk. While the domestic milk brands have repeated quality problems, Weidendorf milk has never had food safety problems and also tastes better than domestic brands; therefore, it is favored by the majority of consumers.

The fourth group focuses on the hot topic of expired food. The core user of this group, user Tahini, published a highly agreeable answer about the popularization of expired food, which triggered a hot debate among internet users. The respondent expressed his views on whether food can still be consumed after the expiration date, saying that the shelf life of food sold on supermarket shelves should be called the best consumption period of food. It is not certain whether or not quality problems will occur after the shelf life, but if the quality of food problems occurs during the shelf life, the producer and seller should be legally responsible. The keyword “liquor” frequently appears because most users mention that liquor does not have a shelf life. The keywords “supermarket” and “milk” appear because supermarket shelves often sell expired milk and yogurt, and some users said they would drink expired milk and yogurt.

The fifth discussion group focuses on food safety problems encountered when eating in restaurants, with keywords such as “merchant,” “complaint”, and “rights protection.” This topic indicates that consumers are now clearer in their attitudes towards food safety problems and are willing to negotiate and complain to solve problems and actively defend their rights. The terms “Consumer Association” and “12315” indicate that users are now going through official channels, such as the China Consumers Association and the Market Supervision Bureau, to address food safety issues.

The hot topics in the five user communities can be divided into three categories: (1) food safety issues that have been exposed by the news media, i.e., the “Laotan pickled cabbage” and “Weidendorf milk” incidents in Groups 1 and 3; (2) food safety science articles, i.e., professional science on food shelf life and cooking oil in Groups 2 and 4; and (3) food safety issues that are encountered in the daily lives of online users, such as the lack of freshness and hygiene of ingredients in take-out and restaurants, and the difficulties consumers face in negotiating, defending their rights and appealing.

(2) Keywords based on TF-IDF algorithm

In order to better filter the words that are irrelevant to the text and keep the keywords with high impact, the TF-IDF (Term Frequency–Inverse Document Frequency) algorithm is used. The TF-IDF algorithm is used to evaluate the importance of a word to a document set or a document in a corpus. Based on the number of occurrences in the text and the document frequency in the whole corpus, the keywords are assigned weights, as shown in Table 5.

According to the TF-IDF algorithm, the keywords with the highest weight in each group are “pickle,” “fatty acid,” “Weidendorf,” “expired,” and “merchant,” which show the topics that the five groups of users are concerned with. The results are similar to the ones obtained by word frequency sorting. The second group of users is most concerned with fatty acids in the popular science about cooking oil since saturated fatty acids and trans fatty acids are harmful to the human body. The core of the discussion of the fourth group of users is about Weidendorf milk, comparing it with Mengniu, Yili, and other national brands.

(3) Key phrase extraction based on TextRank algorithm

The TextRank algorithm is a graph-based text ranking algorithm that filters the deactivated words of each sentence afterword segmentation to obtain a sentence set and a word set. Each sentence is treated as a window, and the window size is set to K. Each word is treated as a node in the PageRank, and the window size is also set to K. There exists an undirected and unweighted edge between any two words corresponding to nodes in each window, and based on this, the importance of each word (node) can be calculated. Once there are several keywords adjacent to each other in the text, the key phrase is formed.

Table 6 shows the keywords and key phrases based on the TextRank algorithm. Group 1 thinks that domestic companies should not treat exported and domestic products differently; they should consider the health of Chinese nationals, and unscrupulous and untrustworthy food companies should be punished. Group 2 is concerned about the issue of whether GM food is harmful while focusing on fatty acids. Group 3 discussed domestic versus imported brands in light of the Weidendorf milk incident. Group 4 expressed that they do not often look at the shelf life of food when they go to the supermarket, and finally, Group 5 thinks that they should complain again when the consumer association does not deal with the food safety problems reported by consumers.

#### 3.3.2. Emotional Characteristics Analysis

In order to analyze the emotional characteristics of netizens under the topic of food safety, Snow NLP, a Python library for Chinese natural language processing, is utilized. The tool is based on a sentiment dictionary, which maps words in a given text to sentiment scores. The main functions include lexical annotation, sentiment analysis, keyword extraction, and text summarization. The sentiment is negative when the sentiment score falls within the interval of [0, 0.5), and positive when it falls within the interval of [0.5, 1].

**Figure 3:**
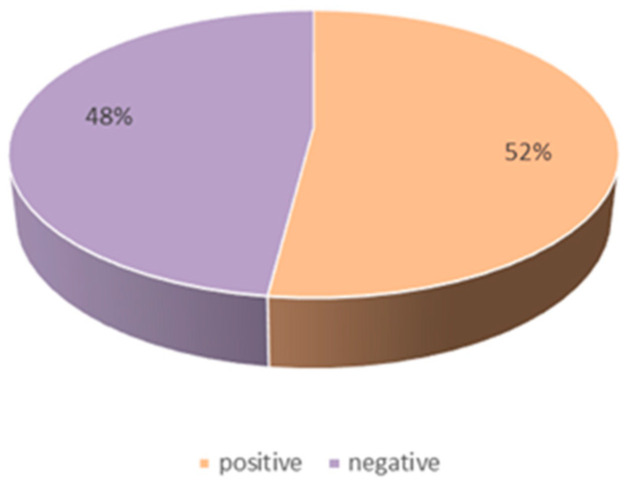
Statistics of the overall emotional value of users.

**Figure 4:**
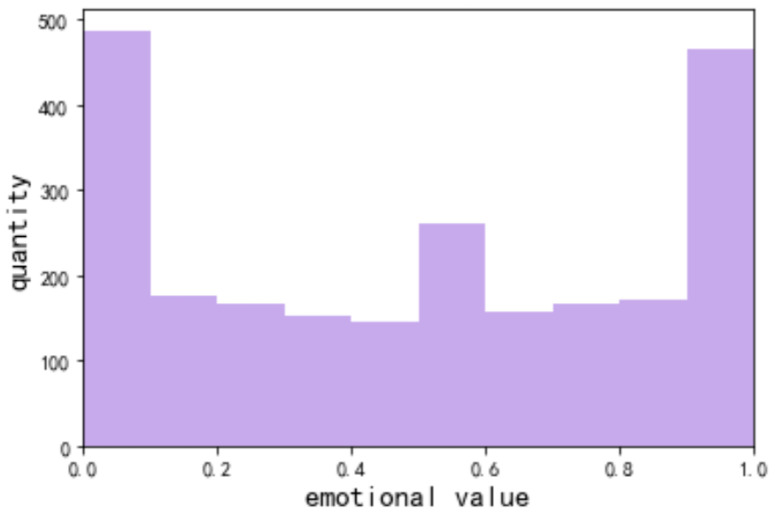
The overall emotional distribution of users.

Figure 3 shows the emotional values of all users, and Figure 4 shows the emotional distribution of users. Under the topic of food safety, the overall positive and negative emotions of users are almost equal, with 48% of users having a negative emotional state and 52% of users having a positive emotional state. The number of people with sentiment scores between 0 and 0.1 and 0.9 and 1 is the largest, which indicates that users are prone to express some extreme (negative or positive) emotions on the topic of food safety.

Table 7 shows the distribution of the sentiment values of the five groups of users that have been analyzed throughout this study thus far. It is found that the overall trend of the sentiment value is extremely low or extremely high, with different values in each group.

Group 1’s focus is on the sanitation scandal of Laotan pickled cabbage. Negative emotions accounted for a large proportion of the users (66.2%), and 35% of users had an emotional value of [0, 0.1], reflecting their extreme dissatisfaction with the incident.

Group 3’s focus is on the topic of CCTV exposure, and they have a relatively large share of positive emotions (71.9%), with a high percentage of users whose emotion value is at (0.9, 1], accounting for 31%. Users mostly hold an understanding and supportive attitude towards Weidendorf milk, believing that Weidendorf milk has been reviewed by numerous regulatory authorities and is of high quality, while food quality and safety are precisely the elements that consumers are most concerned about when selecting products.

Groups 2 and 4 mainly discuss science-based topics, and the proportion of positive and negative emotions is similar, around 50% each. For these science-based topics, there is a clear split in views and opinions, and the proportion of extreme positive and negative emotions is similar at around 20% each.

Group 5 discusses food safety problems encountered during restaurant and take-out dining, and this group has a larger proportion of negative emotions (64.5%), with 43% of users with sentiment values at [0, 0.1], close to half of the users. This result indicates that most users are likely to face food safety problems when eating at restaurants or ordering take-out and feel angry and dissatisfied with their experiences.

The distribution of emotions between the overall user base and the subgroups that we identified shows that there are differences in emotional states between users in each subgroup and in the overall group. The overall number of users in the highly positive and highly negative emotional states is almost the same, showing a divide in overall user experiences and opinions regarding food safety. However, within the grouped data, it is shown that, except for the science topics, users’ emotions tend to be extremely negative or extremely positive in response to food safety topics.

#### 3.3.3. Analysis of Industry Characteristics

**Figure 5:**
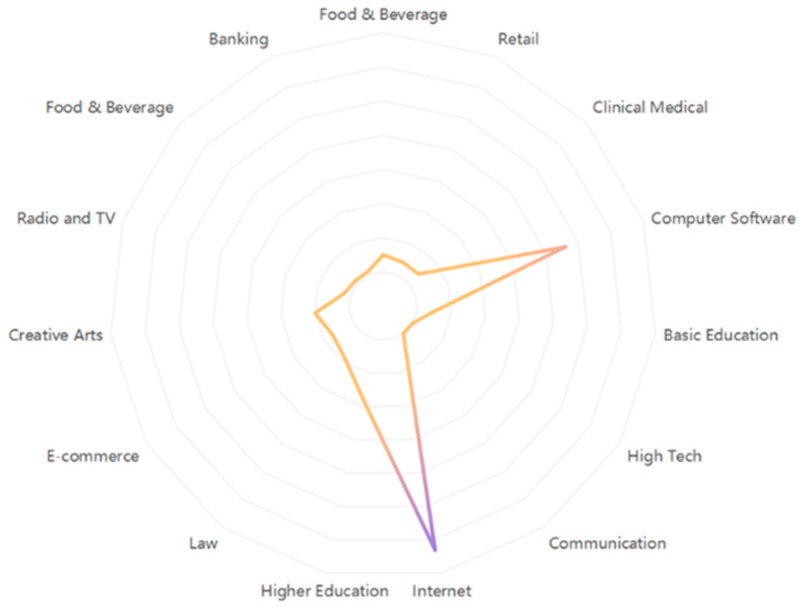
The overall industry characteristics of users on the topic of food safety.

A radar chart, also known as a spider plot, is a chart showing the strength of multidimensional data. It maps the data of multiple dimensions onto the coordinate axis. Based on the personal information of users in each community, the study obtained information about their industries and selected the top 15 industries with the highest number to draw a radar chart. Users on the Internet, computer software, and higher education industries post the most content on the topic of food safety within Zhihu’s platform (see Figure 5). In recent years, there is increasing employment in Internet-related industries, such as those engaged in Internet operation services, application services, information services, and network products. Therefore, more people who are better at using intelligent electronic devices, which likely leads to more activity in online communities, such as Zhihu. 

Table 8 shows the top 10 industries in terms of the number of users within each community based on our community groupings. Although the *Internet* or *computer software* industry ranks first in each group, there are still some differences across the five groups.

The topics discussed in Groups 1 and 3 are hot topics of food safety exposed by CCTV. The industries of their users are widely and evenly distributed, including *banking*, *real estate*, *automotive*, *communications*, and other industries. Clearly, netizens from all walks of life are sharing their comments and opinions on food safety topics.

The topics discussed in Groups 2 and 4 are popular science topics in the field of food safety. Many users in the *catering* industry and the *food and beverage* industry have participated in these discussions. The *catering* industry pays more attention to the evaluation of cooking oils, and the *food and beverage* industry pays more attention to the issue of food shelf life. This indicates that more food safety topics that are discussed on Zhihu are likely to attract users from related fields of employment, which is likely to lead to stronger subject-matter expertise being shared within related Q&A conversations. Users in the catering industry and food and beverage industry can bring more professional answers, help, and gain more knowledge in discussions in the online community.

The topic discussed in Group 5 focuses on whether consumers should protect their rights when food safety problems occur and what are the means and ways to protect their rights. Correspondingly, there are many employees in the *legal* industry in this group, and they help other users from a professional point of view. Netizens in the legal circle believe that when consumers encounter food safety problems, they should have the courage to defend their rights, protect themselves through legal means, and reasonably strive for their rights and interests.

In all, there are differences in the industry characteristics of users among the groups. Hot topics on the Internet for food safety attract the attention of various industries, but under targeted topics, more users from related industries are involved in the discussion.

## 4. Public Opinion Network Analysis

### 4.1. Biterm Topic Model Analysis

The traditional LDA topic model is not effective when dealing with short texts because the words in the text are sparse. BTM is an unsupervised statistical learning model that is proposed for short texts and improves upon the traditional LDA model. BTM reveals the correlation between words through a two-word-based word co-occurrence pattern, which is then applied for topic analysis [34].

**Figure 6:**
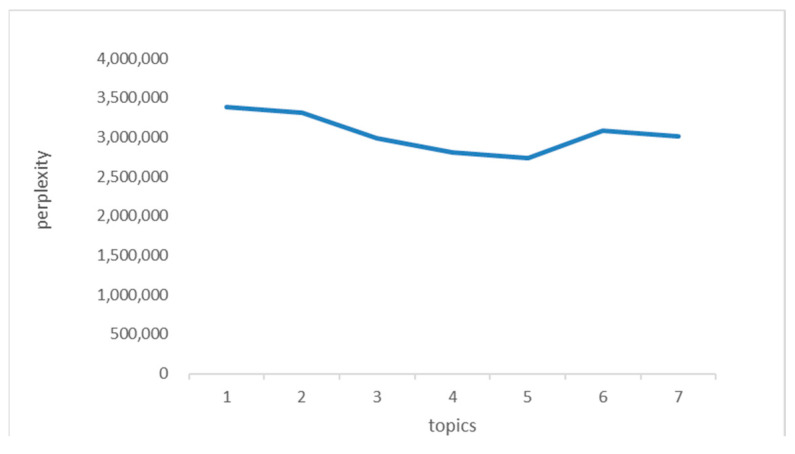
The perplexity of the BTM topic model.

Perplexity is the standard to measure the number of topics selected by natural language models. When the model is more capable of generating complexity, the complexity value is smaller. Figure 6 shows the perplexity under BTM topic model, where the parameters are set as alpha = 0.5, beta = 0.5, *n*_iter = 10, and save_step = 100. This figure shows that the confusion degree is lowest when the number of topics, K, is 5.

Table 9 lists the intensity of each topic, the top eight keywords under the topic, and the intensity of each keyword.

Under the BTM model, five topics are classified according to perplexity, and the topic intensities are 0.67, 0.13, 0.09, 0.08, and 0.02, respectively. The intensity of Topic 1 is much higher than the others.

The keywords such as “merchant,” “shelf life,” and “complaint” in Topic 1 demonstrates users’ concerns about food safety issues such as expired food and Laotan pickled cabbage. When these food safety issues occur, consumers should pursue merchants in a timely manner and use formal means to defend their rights. Topic 2 is about food safety science knowledge. Topic 3 shows the current situation that supermarkets often sell bread, milk, and other expired discounted food. Topic 4 concerns brands with food safety problems, such as Yili, Mengniu, and Yang Guofu, where consumers are prone to long-term rejection of brands that have had food safety problems. Topic 5 discusses whether consumers should apply for refunds, compensation, or even file complaints to fight for their legal rights when food safety problems occur when they dine out or take out.

### 4.2. Semantic Network Analysis 

The subject terms extracted by the BTM model are mainly based on word co-occurrence, where the subject terms are discrete, and the correlation between the subject terms is not considered. The PMI index is applied to measure the correlation between two subject terms (keywords). Table 10 shows the edge weights between keywords based on the point-to-point information index. For example, “CHAQICAIYE” and “Tongyi” have the highest edge weight because CHAQICAIYE is the supplier of pickles for Tongyi instant noodles. Oleic acid is a kind of unsaturated fatty acid, which is an important substance in cooking oil; thus, the edge weight between “Oleic acid” and “unsaturated” is high.

Table 11 shows the keyword degree centrality. The opinion network graph is an undirected graph. Degree centrality represents the number of times that this keyword co-occurs with other keywords, and the weighting degree is the weight of the edge between words calculated by PMI. Among all the keywords, “problem,” “food,” and “time” have the highest degree of centrality and are all high-frequency terms in food safety topics. The keywords “merchant” and “consumer,” as the most important players in the food industry, are also highly relevant to other terms.

**Figure 7:**
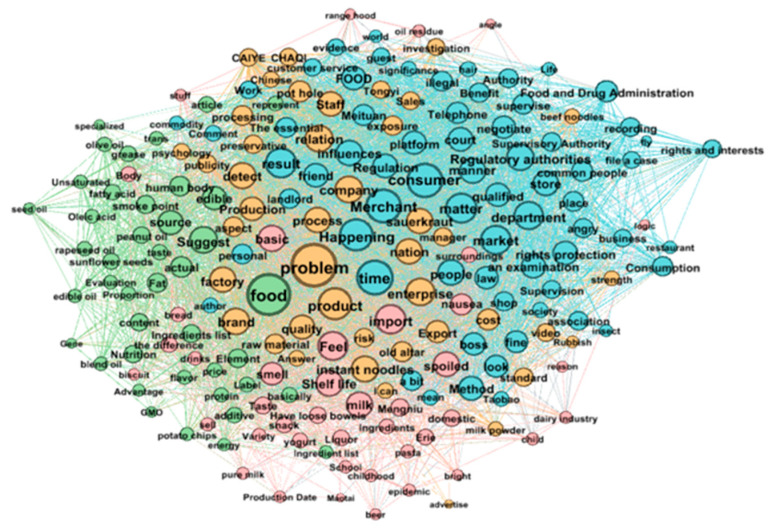
Semantic community structure.

Figure 7 shows the semantic community structure. After using the community detection algorithm to modularize the nodes, the modularity class parameter is obtained, and the semantic network is divided into four communities with nodes of different colors representing different communities. The words within each community are more strongly associated with forming semantic communities, and each community represents a discussion topic. 

Table 12 shows the specifics of the semantic community division formed by the community detection algorithm, including the degree (importance) of each keyword.

Topic 1 focuses on the information available on food packaging, including the shelf life and ingredient list, the best “before date” of the food, and the ingredients and additives in the ingredient list, all of which reflect users’ concerns.

Topic 2 focuses on the participants in the food industry, including consumers, merchants, and regulators. Users express that consumers should use legal means to defend their rights when they encounter food safety problems, while regulators should also increase the frequency and intensity of food safety supervision and punishment.

Topic 3 focuses on the controversial topic of imported and exported food products. Whether it is milk or Laotan pickled cabbage, the quality of imported and exported foods is generally higher than that of domestic foods.

Topic 4 focuses on science topics about the nutritional value of food. Users discuss the ingredients and nutritional content in food, especially caring about the nutritional value of cooking oil.

## 5. Conclusions and Suggestions

### 5.1. Conclusions

Domestic food safety incidents have occurred frequently throughout China, triggering great public concern regarding food safety issues. In order to explore the public’s awareness and help improve the current state of food safety and build a harmonious online community, the hot events under the topic of “food safety” on the Zhihu platform are studied in this research. An overall food safety user network is constructed, where key users in the group are identified based on degree centrality and eigenvector centrality. Further, the user network is divided into multiple communities to analyze community-specific topics and conduct sentiment analyses on the posting within each community.

The TF-IDF and TextRank algorithms are used to determine high-frequency keywords in each community. The python SnowNLP library is used to analyze the sentiment status of users in each community. The BTM model is applied to identify the discussion topics under food safety topics on the Zhihu platform. Further, Gephi is used to map the semantic network and identify the topics through the community segmentation algorithm. Finally, based on the results of user network and opinion network analysis, our conclusions are as follows:

(1) Internet users in the field of food safety form virtual communities with a wide range of diverse topics of concern. It is found that users form distinct community divisions. Most of the users in the community have the same interests. The opinions of the core users in the community have a certain guiding effect on the general users, and the topics concerned by each community are different, which is mainly divided into three aspects. One is the major food safety issues exposed by the official media such as CCTV, which can easily lead to heated discussions among netizens, expressing their emotions and putting forward their own opinions. The other is food safety, a popular science topic under the Zhihu platform. These topics have been widely welcomed by netizens, and users all expressed their appreciation for the popular science experts in the food industry. The third is the food safety problems experienced when dining out and ordering food to go. Users often share and discuss personal experiences on these topics. It is noteworthy that netizens concern more about news reports of failed food safety tests and problems with unclean food encountered in their lives than about actual disease risks, which is consistent with the findings of existing food safety opinion studies [35,36].

(2) The formation of online communities is related to users’ concerns, emotional states, and industry characteristics. It is found that there are significant differences in the hot topics and emotional characteristics of users in different online communities, and users in each online community have the same tendency to pay attention to or feel the same way. The industry differences among groups are reflected in the fact that users are more likely to express their opinions and participate in discussions on topics in which they hold subject-matter expertise.

(3) Consumers’ awareness of food safety is increasing, and awareness of rights is strengthening. Multiple methods are used to classify the text data into topics, among which those about consumer complaints and rights protection are of high intensity. With the growth of social and economic activity, consumers are now more aware of their rights, and their demands tend to be more reasonable and legitimate (i.e., based on facts and experiences). There are more diverse channels of complaint and reporting, such as through 12,315, 12,345, and other complaint hotlines, or online platforms such as takeaway platforms and review platforms. The increased awareness of consumer self-protection plays a good regulatory role for food industry producers and sellers, and the timely resolution of any problems found helps to promote food safety governance.

### 5.2. Suggestions 

#### 5.2.1. To Relevant Departments

(1) Increase food safety supervision to enhance food quality assurance. Held on 27 April 2022, the “national food safety work conference of the market supervision system” emphasized that the food safety defense line should be steadily guarded against the height of politics, with extreme responsibility and with the strength of the whole system and tough violation measures. Therefore, in view of the frequent occurrence of food safety problems in China in recent years, the relevant regulatory authorities should implement the important instructions of General Secretary Xi Jinping on food safety and production safety, enhance the unity and professionalism of food safety supervision, improve the level and capacity of food safety supervision, solve the concerns and confusion of the general public, and guarantee “safety on the tip of the tongue”.

(2) Enhance the quality and safety of local food and improve the influence of national brands. According to the Laotan pickled cabbage topic, the phenomenon of “double standards” in food safety has been widely discussed recently. The CHAQICAIYE Company treats domestic consumers and exported consumers differently with different quality products, disregarding the health of domestic consumers. Similar issues have led to more and more domestic consumers preferring imported products with quality assurance guarantees, safety, and security. Such preference towards imported goods is not a good sign for domestic food production and the related economical effects.

As early as 2016, China has been promoting and encouraging the “same line, same standard, same quality” project for domestic and foreign products. Companies should produce domestic and export goods according to the same standard on the same production line so that products supplied to the domestic market meet the same quality standards as those in the international market. Domestic manufacturers and sellers should improve the quality control and supervision of domestic products to meet consumers’ expectations as much as possible and continuously improve the brand quality and influence of national brands.

(3) Strengthen the awareness of consumers’ rights so that consumers can better defend and protect such rights. With the development and growth of China’s market economy, the food industry has seen an increase in the number of merchants, which has to a certain extent, led to an increase in violations, and consumers are prone to encounter infringements. Firstly, information related to the protection of rights should be better publicized throughout the media to help consumers establish a reasonable and legal awareness of their rights. Secondly, the popularization of the Consumer Rights law and other related laws would help to eliminate the legal blind spots for consumers and therefore protect their rights as needed. Finally, policy needs to urge the relevant departments to actively accept and resolve food safety disputes, effectively solve problems for the people, and encourage consumers to report food quality/safety problems.

#### 5.2.2. To Web Platforms

(1) Attention should be paid to the cluster effects within the online community, which can help to promote the healthy development of the network environment. It is found that the topic of food safety shows a strong cluster effect, with multiple virtual communities that have unique interests and emotional tendencies. The emergence of online communities makes it easy for users to find like-minded partners and discuss hot issues, but clusters can lead to extreme emotional tendencies, exaggerate real-life conflicts, and generate and spread irrational emotions (e.g., echo chambers). In order to purify the current situation of online platforms and promote the healthy development of online environments, platform managers should pay attention to the cluster effect, which would likely help in controlling the spread of false information and radical emotions [37]. 

(2) Monitor the statements of key community users to effectively guide the platform’s public opinion. The statements of key users in the online community have a strong guiding effect on other general users and the overall discussions. Further, given that key network users cannot always guarantee the accuracy and reliability of their published information, this could lead to the spread of online rumors and false information. Online platforms should establish a more complete and effective network information supervision system, monitor the content posted by key users in the community as much as possible, reduce the spread of false information, and guide positive platform public opinion orientation.

(3) Strengthen the science of food safety knowledge to help raise food safety awareness. According to the data, most users of the Zhihu platform are interested in food safety knowledge and respond to comments under topics related to food science and nutrition. Online platforms can take advantage of users’ positive psychology and sense of knowledge to enhance food safety knowledge. 

There are some limitations in the research. For example, only the Zhihu platform is selected, and the number of Internet users who are concerned about food safety topics is not comprehensive enough. In future research, it would be interesting to apply the methodology used in this paper to analyze other platforms where food safety issues are likely discussed, such as Yelp. In addition, the depth and breadth of data collection can be further expanded, such as by selecting more relevant topics in this field for research and lengthening the period of data collection.

## Figures and Tables

**Table 1:** The statistics of degree centrality for the top 10 users.

Number	ID	Degree	Weighted Degree
1	Tahini	1099	7254
2	Qian Cheng	462	1983
3	milk	404	470
4	Wang SH	386	395
5	Dongjun	386	395
6	Xiao Chubai	386	395
7	boy sleep	378	403
8	Zhihu user 0xyqjw	371	395
9	Zhihu user wp94Wr	370	395
10	Socra bottomless	297	318

**Table 2:** Statistics of edge weight in the user relationship graph.

Number	ID	ID	Edge Weight
1	Qian cheng	Lyodi	95
2	Tahini	Sun mulin	85
3	Tahini	Bubble	67
4	Tahini	Muzixi	32
5	Tahini	Meng dong	30
6	Qian cheng	Levin spirit	27
7	Qian cheng	Shunsungbai	27
8	Qian cheng	Self-discipline set me free	20
9	Milk	Eazy	6
10	Socra bottomless	Fairy feather	3

**Table 3:** Statistics of users’ eigenvector centrality.

Number	ID	Eigenvector Centrality
1	Rice grains	1
2	Suifeng	0.667673
3	Nicole	0.667673
4	Kratos	0.667673
5	Melon eaters	0.657583
6	Boy sleep	0.572291
7	Hello ha	0.572291
8	Summer solstice	0.562201
9	Deep cooling	0.562201
10	This is not a vest	0.427709

**Table 4:** Hot topics based on word frequency.

Importance	Group 1	Group 2	Group 3	Group 4	Group 5
1	Pickle	Fatty acid	Milk	Expired	Merchant
2	Company	Rapeseed oil	Import	Shelf life	Complaint
3	Problem	Gmo	Mengniu	Food	Consumer association
4	Food	Peanut oil	Yili	Liquor	Compensation
5	China	Olive oil	China	Milk	Manner
6	Us	Smoke point	Brand	Supermarket	Consumer
7	Own	Gene	Domestic	Feel	Rights protection
8	Abroad	Blend oil	Dairy industry	Ingredients list	Direct
9	Food safety	Fat	Association	Smell	12,315
10	Pot hole	Grease	Delicious	Merchant	Market

**Table 5:** Keywords and their weights based on the TF-IDF Algorithm.

Group 1	Group 2	Group 3	Group 4	Group 5
Pickle (0.3602)	Fatty acids (0.0978)	Weidendorf (0.1767)	Expired (0.3391)	Merchant (0.1000)
Laotan (0.1257)	Smoke point (0.0907)	Milk (0.1400)	Shelf life (0.1578)	Consumer association (0.0730)
Instant noodles (0.0885)	Peanut oil (0.0825)	Mengniu (0.1091)	Food (0.0644)	Complaint (0.0677)
Food (0.0451)	Canola oil (0.0758)	Import (0.0955)	White wine (0.0583)	Meituan (0.0626)
Security (0.0439)	Oleic acid (0.0685)	Yili (0.0870)	Ingredient list (0.0490)	Respondent (0.0584)
Pot hole (0.0430)	Olive oil (0.0666)	Dairy (0.0702)	Milk (0.0467)	12,315 (0.0459)
Chaqi (0.0367)	Blending oil (0.0651)	National production (0.0577)	Supermarket (0.0431)	Compensation (0.0443)
Aboard (0.0340)	Genetically modified (0.0638)	Good to drink (0.0551)	Clinical (0.0384)	Yang guofu (0.0417)
Company (0.0335)	Sunflower Seeds (0.0628)	Brand (0.0482)	Yogurt (0.0263)	Shopkeeper (0.0370)
Vegetable industry (0.0306)	Transgene (0.0529)	Taste (0.0346)	Respondent (0.0262)	Defend the right (0.0353)
315 (0.027 merchant 5)	Linolenic acid (0.0443)	Association (0.0339)	Thanks (0.0257)	Takeaway (0.0338)
Exposure (0.0268)	Oleic acid (0.0423)	Quality (0.0280)	Taste (0.0252)	Fda (0.0264)

**Table 6:** Keywords and key phrases based on TextRank algorithm.

Group	Keywords	Key Phrases
Group 1	Pickle (0.01965) Company (0.00669) Problems (0.00615) Laotan (0.00454) China (0.00451) Food safety (0.00333)	Chinese people, Laotan pickled Cabbage, Food safety issues Food companies, Should be fined
Group 2	Canola oil (0.00835) Genetically modified (0.00711) Fatty acids (0.00666) Blended oils (0.00654) Fats and oils (0.00523) Olive oil (0.004669)	Eat genetically modified food, Olive oil smoke point
Group 3	Milk (0.015135) Weidendorf (0.011794) China (0.008535) Domestic (0.007850) Problem (0.007818) Brand (0.006899)	Chinese brand, Buy imported milk, Weidendorf milk, Imported milk, Domestic milk, Chinese brand Chinese milk, Domestic milk
Group 4	Expired (0.015493) Shelf life (0.007591) Food (0.007492) Milk (0.005094) Supermarket (0.004627) Stuff (0.003772)	Expired food, buying, Going to the supermarket, Expired milk, Food shelf life, Do not look
Group 5	Merchants (0.009681) complaints (0.007066) Consumer association (0.004578) Meituan (0.004517) Compensation (0.004304) Attitude (0.004250)	Merchant’s attitude, Complaints against consumer associations, Shopkeeper attitude

**Table 7:** User group sentiment value statistics.

Emotional Value	Group 1	Group 2	Group 3	Group 4	Group 5
[0,0.1]	34.74%	17.58%	6.75%	17.75%	42.50%
(0.1, 0.2]	9.42%	9.09%	4.68%	7.70%	6.25%
(0.2, 0.3]	7.47%	9.09%	6.49%	8.08%	3.75%
(0.3, 0.4]	9.09%	4.85%	3.90%	7.23%	5.25%
(0.4, 0.5]	5.52%	9.09%	13.77%	6.67%	15.00%
(0.5, 0.6]	4.87%	5.45%	6.75%	9.01%	3.50%
(0.6, 0.7]	7.47%	6.67%	7.53%	7.42%	4.00%
(0.7, 0.8]	4.87%	5.45%	11.43%	7.98%	3.75%
(0.8, 0.9]	3.57%	7.88%	8.05%	8.83%	4.25%
(0.9, 1.0]	12.99%	24.85%	30.65%	19.34%	11.75%

**Table 8:** Industry characteristics of users in each of the five groups.

Group 1	Group 2	Group 3	Group 4	Group 5
Internet	Computer Software	Computer Software	Internet	Internet
Higher Education	Internet	Internet	Computer Software	Legal
Computer Software	Catering	Higher Education	Food and beverage industry	Computer Software
Electronic Games	Securities Investment	Creative Arts	Higher Education	Dining
Basic Education	Higher Education	E-Commerce	Creative Arts	Creative Arts
Banking	High-tech	Legal	Basic Education	Clinical Medicine
E-commerce	Finance	Clinical Medicine	E-commerce	E-Commerce
Real Estate	Real Estate and Construction	Automotive	Retail	Higher Education
High-tech	Non-Profit Organizations	Plastics Industry	Law	Retail
Machinery and Equipment	Public Services	Communication	Radio and TV	Radio and TV

**Table 9:** Keyword distribution of BTM topic model.

Topic	Keywords (Keyword Intensity)	Topic Intensity
Topic 1	Issue (0.008532) Expired (0.007945) Pickle (0.007840) Merchant (0.007075) Food (0.007002) Shelf life (0.006061) Complaint (0.004775) Knows (0.004747)	0.672764
Topic 2	Fatty Acids (0.006340) Oleic Acid (0.005245) Smoke Point (0.005086) Canola Oil (0.005023) Snacks (0.004896) Protein (0.004531) Content (0.004102) Olive Oil (0.003975)	0.128702
Topic 3	Supermarket (0.011500) Food (0.005515) Product (0.004358) Shelf Life (0.004183) Bread (0.003986) Discount (0.003964) Advent (0.003637) A box (0.003222)	0.091485
Topic 4	Yili (0.005115) Problem (0.004670) Yang Guofu (0.004272) Mengniu (0.004132) Zhihu (0.003710) Import (0.003406) A bowl (0.003383) Thanks (0.003359)	0.084866
Topic 5	Merchant (0.014048) Meituan (0.007407) Complaint (0.005802) Refund (0.005729) Customer Service (0.005291) Store (0.003539) Compensation (0.003320) Attitude (0.003320)	0.022184

**Table 10:** Edge weights of co-occurring words.

Number	Keyword 1	Keyword 2	Edge Weight
1	CHAQICAIYE	Tongyi	7.97728
2	Oleic acid	Unsaturated	7.61471
3	Oleic acid	Evaluation	7.199672
4	Recording	Survey	6.199672
5	Recording	Industry and Commerce	6.199672
6	Pot hole	Raw materials	6.070389
7	Problem	Milk industry	2.061827
8	Food	Problem	1.61252
9	Food	Shelf life	0.843474
10	Food	Pickle	0.840472
11	Problem	Additives	0.592342
12	Problem	Genetically Modified	0.476865

**Table 11:** The statistics of keyword degree centrality.

Sequence	Keyword	Degree	Weighted Degree
1	Problem	149	260.095832
2	Food	142	432.464567
3	Time	114	330.291005
4	Consumer	112	333.966639
5	Merchant	110	252.119566
6	Product	103	285.121101
7	Result	94	267.415715
8	Matter	94	264.3713
9	Import	92	194.261246
10	Market	91	287.963706
11	Feel	90	134.312876
12	Department	87	306.098726
13	Relation	82	289.869523
14	Suggest	82	242.560343
15	Brand	81	247.497752
16	Regulatory authorities	80	259.365104

**Table 12:** Semantic community division of the topic of food safety.

Topic 1	Degree	Topic 2	Degree	Topic 3	Degree	Topic 4	Degree
Shelf life	80	Time	114	Problem	149	Food	142
Milk	78	Consumer	112	Product	103	Suggest	82
spoilage	75	Merchant	110	Import	92	Source	73
Taste	68	Market	91	Company	84	Body	54
Ingredient List	45	Regulators	81	Testing	83	Fat	50
Taste	45	Attitude	77	Pickle	81	Ingredients	49
Mengniu	45	Court	73	Brand	81	Content	46
diarrhea	38	Stores	73	Country	77	Smoke Point	46
snacks	37	rights protection	71	Quality	74	Nutrition	43
additives	37	Regulations	71	Staff	74	Peanut oil	39
environment	36	Inspection	70	Production	72	Oils and Fats	36
white wine	36	Catering	70	Manufacturers	70	Article	36
Author	35	Qualified	69	Export	63	Answers	35
Distinction	35	Platform	68	pot hole	62	Fatty acids	35
Body	33	Consultation	67	Disgusting	61	price	33
Ingredient	33	Legal	65	Instant Noodles	59	Tags	33
Protein	32	Fines	62	Laotan	58	canola oil	30
Yogurt	32	Meituan	59	Exposure	53	olive oil	30

## Data Availability

The data presented in this study are available on request from the corresponding author.
